# Spiropyran-Based Soft Substrate with SPR, Anti-Reflection and Anti-NRET for Enhanced Visualization/Fluorescence Dual Response to Metal Ions

**DOI:** 10.3390/ma16103746

**Published:** 2023-05-15

**Authors:** Yuebo Jin, Florian Ion Tiberiu Petrescu, Yuan Wang, Xin Li, Ying Li, Gang Shi

**Affiliations:** 1 The Key Laboratory of Synthetic and Biotechnology Colloids, Ministry of Education, School of Chemical and Material Engineering, Jiangnan University, Wuxi 214122, China; 2 Department of Mechanisms and Robots Theory, Bucharest Polytechnic University, 060042 Bucharest, Romania

**Keywords:** bioinspired, spiropyran, fluorescence, visualization, ion detection

## Abstract

The photoluminescence of modified spiropyran on solid surfaces is poor, and the fluorescence intensity of its MC form is weak, which affects its application in the field of sensing. In this work, a PMMA layer containing Au nanoparticles and a spiropyran monomolecular layer are coated on the surface of a PDMS substrate with inverted micro-pyramids successively by means of interface assembly and soft lithography, and the overall structure is similar to insect compound eyes. The anti-reflection effect of the bioinspired structure, the SPR (surface plasmon resonance) effect of the Au nanoparticles and the anti-NRET (non-radiation energy transfer) effect of the PMMA isolation layer raise the fluorescence enhancement factor of the composite substrate vs. the surface MC form of spiropyran to 5.06. In the process of metal ion detection, the composite substrate can achieve both colorimetric and fluorescence response, and the detection limit for Zn^2+^ can reach 0.281 μM. However, at the same time, the lack of the ability to recognize specific metal ions is expected to be further improved by the modification of spiropyran.

## 1. Introduction

Spiropyrans have attracted much attention because of their ability to bind various metal ions and provide a unique spectral response to each metal ion [1,2,3]. As a typical representative of a photochromic molecule, spiropyran can convert to the colored merocyanine (MC) form due to C-O bond breaking under UV light induction and can return to its colorless initial state (SP) through a ring-closure reaction under visible light or heating stimulation [4]. The negatively charged phenoxy group in MC can provide complex sites for metal ions, causing the absorption spectrum and fluorescence spectrum of spiropyran to change when spiropyran interacts with metal ions [5,6]. As a result, spiropyran can be used in colorimetry and fluorescence sensing for metal ions. However, the spiropyran molecule has some shortcomings, such as easy photodegradation leading to poor fatigue resistance [7,8], easy aggregation of the MC form leading to weak fluorescence and inability to undergo the ring-closure reaction [9], which limit its practical application in the field of sensing.

An effective way to solve these problems is to immobilize spiropyran on other supports (polymer chains, nanoparticles, etc.) through covalent bonds [10,11]. The supports will affect the switching dynamics of spiropyran to a large extent, avoid spiropyran aggregation and improve its photostability [12], switchability and processability. According to the dispersed form of spiropyran, the immobilization modes can be divided into two types: The first type is to copolymerize spiropyran monomers with other monomers to form a copolymer. For example, Locklin et al. prepared copolymers of methacrylated spiropyran and methyl methacrylate by atom transfer radical polymerization. The ester group on the main chain of the polymer served as an additional coordinative group for metal ions, and the copolymer showed a unique colorimetric response to different metal ions including Cu^2+^, Fe^2+^, Zn^2+^, Co^2+^ and Ni^2+^ [13]. However, the dense polymer network not only prolongs the ring-opening response time of spiropyran but also makes it difficult for metal ions to desorb from the polymer system, which is bad for cyclic performance. The second type is to fix spiropyran on the surface of the support by interface modification. For example, Maclachlan et al. modified spiropyran on a chiral nematic mesoporous organosilica (CNMO) surface with the help of an aminosilylation reagent. The mesoporous properties of the substrate provided a large number of attachment sites for spiropyran. As a result, the substrate exhibited excellent photochromic properties and colorimetric responses to Zn^2+^, Cu^2+^, Ni^2+^ and Sn^2+^ [14]. However, spiropyrans modified on the surface of the support are usually in monomolecular layer forms, and their colorimetric or fluorescence sensing signals are weak. Thus far, trace detection of metal ions cannot be realized by using modified spiropyran on a substrate. Therefore, improving the spectral responsiveness of modified spiropyran on the support surface is the key to expand the application of spiropyran in metal ion sensing.

In our previous work, a Si_ph_/TiO_2_/PMMA-SP/Au@SiO_2_ substrate was fabricated and the fluorescence of spiropyran was effectively enhanced [15]. Introducing noble metal nanoparticles such as Au nanoparticles (AuNRs) into spiropyran-based systems has attracted a great deal of interest in optical and plasmonic devices. There is exciton–plasmon coupling between the spiropyran molecules and the Au nanoparticles, which will enhance the fluorescence of spiropyran. Meanwhile, the localized surface plasmon resonance (LSPR) peak will shift with the photoswitching of spiropyran molecules [16]. What is more, the high absorptivity of AuNRs to UV light could improve the SP-to-MC conversion for spiropyran [17]. For example, Mahdavian et al. show that the conjugation of the MC form of spiropyran with Au nanoparticles enhanced the photogeneration of reactive oxygen species (ROS) [18].

Here, spiropyrans are modified onto the surface of a PMMA membrane containing Au nanoparticles. The support below the PMMA is PDMS with a micron-scale inverted pyramid structure, and the whole structure is defined as PDMS/PMMA-Au/SP. The PDMS/PMMA-Au/SP has a structure similar to the compound eyes of insects, and its large specific surface area and low reflectivity can provide more attachment sites and higher light absorption efficiency for spiropyran, which is helpful to improve the fluorescence intensity of the open-ring MC form of spiropyran. In the meantime, the electromagnetic field effect of Au nanoparticles can further enhance the fluorescence intensity of MC [19,20]. Compared with our previous work [15], the PDMS/PMMA-Au/SP substrate is more flexible, and its original color is white, while the color of the Si_ph_/TiO_2_/PMMA-SP/Au@SiO_2_ substrate was black. The white color of the PDMS/PMMA-Au/SP will not influence the visual identification of metal ions. The novel PDMS/PMMA-Au/SP substrate was successfully applied to detect trace amounts of metal ions.

## 2. Materials and Methods

### 2.1. Reagents and Materials

Monocrystalline silicon (Si) was purchased from GRINM Semiconductor Materials Co., Ltd. (Shandong, China). 2,3,3-Trimethylindolenine was purchased from J&K Scientific Co., Ltd. (Beijing, China). 2-Butanone, piperidine methanol and chloroform-d_1_ were purchased from Sinopharm Chemical Reagent Co., Ltd. (Shanghai, China). 3-Iodopropanic acid was purchased from TCI (Shanghai) Chemical Industry Development Co., Ltd. (Shanghai, China). Dichloromethane, 2-hydroxy-5-nitrosalicylaldehyde, potassium hydroxide, sodium citrate dihydrate, tert-butyldiphenylchlorosilane (TBDS), ethanol, 1-(3-dimethylaminopropyl)-3-ethylcarbodiimide hydrochloride (EDC·HCl) and dimethyl sulfoxide-d_6_ were purchased from Shanghai Titan Scientific Co., Ltd. (Shanghai, China). Sylgard 184 polydimethylsiloxane (PDMS) was purchased from Dow Corning Co., Ltd. (Midland, TX, USA). Trichloro(1H, 1H, 2H, 2H-perfluoro-octyl)silane, chloroauric acid and polymethyl methacrylate (PMMA, M_w_ = 97,000, M_n_ = 48,000) were purchased from Sigma Aldrich (Shanghai, China) Trading Co., Ltd. (Shanghai, China). (3-Aminopropyl)trimethoxysilane (ATMS) was purchased from Energy Chemical (Shanghai, China).

### 2.2. Synthesis of 3-(3′,3′-Dimethyl-6-nitrospiro[chromene-2,2′-indolin]-1′-yl) Propionic Acid (SP-COOH)

SP-COOH was synthesized according to the literature reports [21].

(1) 2,3,3-Trimethylindolenine (30.59 mmol, 4.9 mL) and 3-iodopropanic acid (30.17 mmol, 6.03 g) were dissolved in 10 mL 2-butanone. The mixture was heated at 100 °C for 5 h under nitrogen and under reflux. The precipitate was suspended in 50 mL H_2_O and washed three times with 50 mL dichloromethane. The organic layers were combined and washed three times with 25 mL water. The aqueous layers were combined and filtered. The solvent was removed by reduced-pressure rotary evaporation to give 1-(2-carboxyethyl)-2,3,3-trimethyl-3H-indol-1-ium iodide as a pale-yellow solid (8.26 g, 22.94 mmol, 76.0% yield). ^1^H NMR (400 MHz, CDCl_3_, Me_4_Si) δ 7.97–8.03 (m, 1H), 7.84–7.89 (m, 1H), 7.58–7.64 (m, 2H), 4.67 (t, J = 6.8 Hz, 2H), 3.00 (t, J = 6.8 Hz, 2H), 2.89 (s, 3H), 1.55 (s, 6H).
(2) 1-(2-Carboxyethyl)-2,3,3-trimethyl-3H-indol-1-ium iodide (6.66 mmol, 2.39 g), 2-hydroxy-5-nitrosalicylaldehyde (6.67 mmol, 1.12 g) and piperidine (8.02 mmol, 0.8 mL) were dissolved in 10 mL 2-butanone. The mixture was heated at 100 °C for 3 h under nitrogen and under reflux. The reaction mixture was cooled to room temperature overnight, yielding the raw product as a yellow precipitate, which was filtered, washed with 2-butanone (10 mL) and cold methanol (10 mL) and dried under high vacuum to yield SP-COOH as a bright yellow-green solid (1.80 g, 4.74 mmol, 71.2% yield). ^1^H NMR (400 MHz, DMSO-d6, Me_4_Si) δ 12.19 (s, 1H), 8.21 (d, J = 2.8 Hz, 1H), 8.01 (dd, J = 2.8 Hz, J = 2.8 Hz, 1H), 7.22 (d, J = 10.4 Hz, 1H), 7.11–7.14 (m, 2H), 6.87 (d, J = 8.8 Hz, 1H), 6.80 (t, J = 14.8 Hz, 1H), 6.67 (d, J = 7.6 Hz, 1H), 5.98 (d, J = 10.4 Hz, 1H), 3.34–3.53 (m, 2H), 2.41–2.61 (m, 2H), 1.19 (s, 3H), 1.08 (s, 3H).

### 2.3. Fabrication of PDMS/PMMA-Au/SP Substrate

Fabrication of the PDMS substrate containing inverted pyramid microstructures: First, the anisotropic etching property of monocrystalline Si in KOH solution was used to obtain the Si template with a micro-pyramid structure on the surface after etching for 30 min [22,23,24]. Then, the Si template underwent hydrophobic treatment with trichloro(1H, 1H, 2H, 2H-perfluoro-octyl)silane to facilitate the subsequent stripping of the PDMS. After that, the mixture of PDMS prepolymer and curing agent was poured on the hydrophobic-treated Si template and cured at 68 °C for 3 h [25,26]. After curing, the PDMS was cooled to room temperature and peeled off to obtain a PDMS substrate with inverted micro-pyramids.

Fabrication of the PDMS/PMMA-Au substrate: First, a 20 mL aqueous solution of chloroauric acid (0.01 wt%) was added to a round-bottomed flask and boiled under stirring and reflux. Then, a 0.14 mL aqueous solution of sodium citrate (1 wt%) was quickly added to the boiling solution, and the solution continued to boil for 30 min. The solution was then cooled to room temperature and centrifuged to obtain Au nanoparticles [15]. Subsequently, 0.0455 g of PMMA was dissolved in 20 g of dichloromethane, and 4.2 × 10^−4^ g of Au nanoparticles was added into the above solution. Gold nanoparticles were dispersed evenly through ultrasonic dispersion. After sealing, the mixture was stirred at room temperature for 5 h. Finally, a 50 μL mix solution of PMMA and Au was spin-coated on the surface of PDMS; the spin-coating speed and time were 4500 r/min and 30 s, respectively. After spin-coating, the PDMS was dried in an oven at 60 °C for 1 h to obtain the PDMS/PMMA-Au substrate.

TBDS functionalization of the PDMS/PMMA-Au substrate: First, the dried PDMS/PMMA-Au substrate was treated with oxygen plasma for 5 min, and a large number of hydroxyl groups were attached to its surface. The treated substrate was then placed in a 20 mL ethanol solution of 100 μL tert-butyldiphenylchlorosilane (TBDS) and allowed to stand at room temperature for 30 min to modify the TBDS monomolecular layer on the surface of the substrate. Finally, the substrate was thoroughly washed with ethanol.

Fabrication of the PDMS/PMMA-Au/SP substrate: First, the PDMS/PMMA-Au substrate modified with TBDS was placed in an ethanol solution of ATMS, and then left to stand for 30 min at room temperature to modify the ATMS monolayer on the surface. The substrate was then fully washed with ethanol. After that, the ATMS-modified substrate was placed into a 20 mL ethanol solution containing 35 mg EDC and 5 mg SP-COOH and left for 3 h at room temperature in the dark, and the spiropyran functionalization was completed on the substrate surface. After washing thoroughly with ethanol, the PDMS/PMMA-Au/SP composite substrate was obtained. 

### 2.4. Characterization

^1^H NMR spectroscopy was conducted on a Bruker AVANCE Ⅲ HD 400 MHz spectrometer operating at 400 MHz. SEM and EDS testing of the substrate was performed on an S-4800 (Hitachi, Ltd., Tokyo, Japan). The absorption spectra and the transmission spectra were recorded on a UV-3600 plus ultraviolet–visible–near-infrared spectrophotometer (SHIMADZU Co., Ltd., Kyoto, Japan) with an integrated sphere attachment. The contact angle test was performed on an OCA 40 optical contact angle measuring instrument (Beijing Eastern-Dataphy Instruments Co., Ltd., Beijing, China). The photoluminescence spectra were recorded on an FS5 fluorescence spectrometer (Edinburgh Instruments, Edinburgh, UK). The fluorescence lifetime was recorded on a Lifespec Ⅱ ultrafast time-resolved fluorescent lifetime spectrometer (Edinburgh Instruments).

## 3. Results and Discussion

### 3.1. Fabrication Process and Morphology Characterization of PDMS/PMMA-Au/SP Substrate

**Figure 1:**
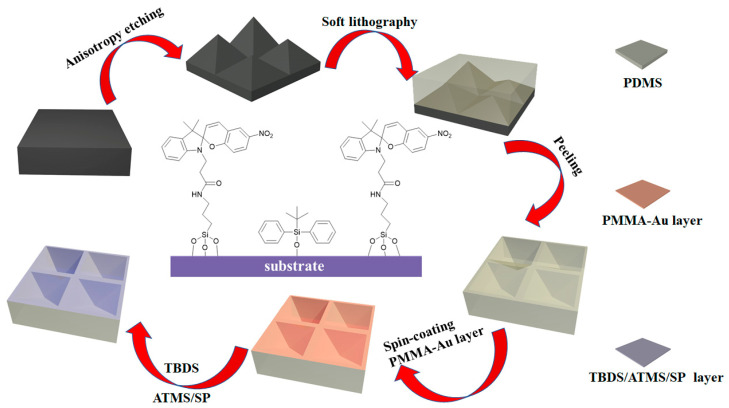
Schematic diagram of the preparation process of PDMS/PMMA-Au/SP substrate.

**Figure 2:**
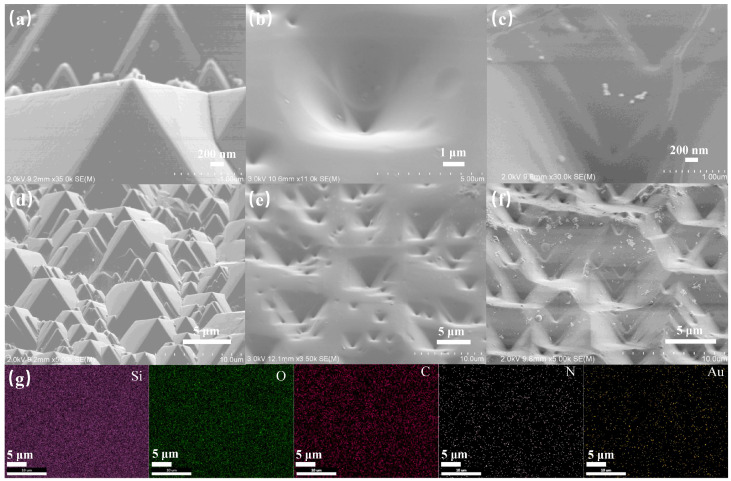
SEM images of (**a**,**d**) Si pyramid array; (**b**,**e**) PDMS surface with inverted pyramid structure; (**c**,**f**) PDMS/PMMA-Au; (**g**) EDS maps of PDMS/PMMA-Au/SP.

The fabrication process of the PDMS/PMMA-Au/SP substrate is shown in Figure 1. Firstly, a Si substrate containing micro-pyramid arrays was obtained by alkali anisotropic etching on a planar Si surface based on the much lower etching rate of plane (111) than plane (100) of monocrystalline Si in KOH solution [27]. The average height of the micro-pyramid was 5 μm, and the angle between the side and the plane was about 54.7°, as shown in Figure 2a,d. Then, the mixture of PDMS prepolymer and curing agent was poured on the Si substrate surface, and the PDMS substrate with inverted pyramid structure was obtained after curing. As shown in Figure 2b,e, the inverted micro-pyramids on the surface of the PDMS complemented the pyramid structure on the Si substrate surface, indicating that the PDMS substrate was successfully impressed with the Si substrate. Compared with the planar structure, this kind of PDMS substrate not only has a larger specific surface area but also has excellent anti-reflection ability, which is beneficial to improve the absorption efficiency of light and enhance the isomerization efficiency and excitation efficiency of spiropyran. After that, PMMA films containing Au nanoparticles were coated on the surface of PDMS. The PMMA, as a dielectric layer, could effectively reduce the probability of direct contact between Au nanoparticles and spiropyran molecules and reduce the non-radiative energy transfer between Au and spiropyran molecules [28,29,30]. In addition, tert-butyldiphenylchlorosilane (TBDS) was modified on the surface of PMMA by reaction of the Si-Cl bond and the hydroxyl group. The modification of TBDS containing rigid groups onto the surface of PMMA could increase the distance between spiropyran molecules, providing enough free space for the isomerization of spiropyran [31]. Finally, (3-aminopropyl)trimethoxysilane (ATMS) was modified on the free sites of the PMMA surface by reaction of the Si-OCH_3_ bond and the hydroxyl group. The decoration of spiropyran on the substrate was achieved by the reaction of the carboxyl group on SP-COOH with the amino group of ATMS modified onto the PMMA surface [14]. Figure 2c,f show the morphology of the PDMS/PMMA-Au/SP substrate, whose overall structure is similar to that of insect compound eyes. Figure 2e shows the EDS spectrum of the PDMS/PMMA-Au/SP substrate, where elements are evenly distributed on the surface of the substrate, further proving that the preparation of the composite substrate was successful.

### 3.2. Molecular Modification of PDMS/PMMA-Au/SP Substrate Surface

**Figure 3:**
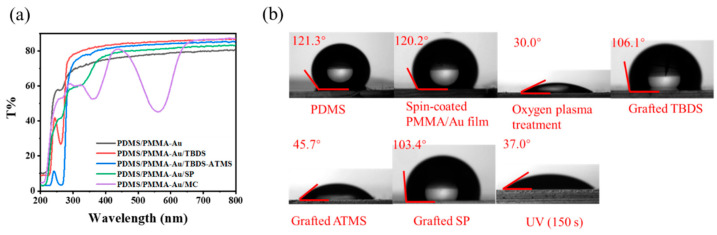
(**a**) UV–Vis transmission spectra of PDMS/PMMA-Au surface grafted with different functional molecules. (**b**) Contact angle changes of the PDMS/PMMA-Au surface after grafting different functional molecules.

In order to further prove the successful modification of functional molecules on the surface of the composite substrate, UV–Vis transmission spectra were obtained for samples involved in the modification process, as shown in Figure 3a. Firstly, two characteristic peaks appeared at 250 nm and 365 nm on the composite substrate surface after modification with TBDS. After modification with ATMS, the characteristic peak at 365 nm disappeared. Finally, the characteristic peak at 250 nm disappeared, and two peaks appeared at 265 nm and 340 nm after the graft of spiropyran. After UV irradiation, the transmission spectrum of the spiropyran-modified composite substrate showed a strong characteristic peak at 550 nm [32], indicating that spiropyran isomerized from the closed-ring SP form to the open-ring MC form, which proved that the preparation of the composite substrate was successful.

Figure 3b shows the variation of the surface contact angle during sample preparation. Both PDMS and PMMA are hydrophobic polymers, so the contact angle of the PDMS substrate and the PDMS/PMMA-Au substrate surface was about 120° without obvious change. After being treated with oxygen plasma, a large number of hydroxyl groups were generated on the surface of PDMS/PMMA-Au, and the contact angle reached 30°. The substrate showed a certain hydrophilicity. After TBDS modification, the contact angle of the PDMS/PMMA-Au surface reached 106.1°. This is because the modified TBDS contains a large number of benzene rings, which makes the surface of the sample show a certain hydrophobicity. After grafting with ATMS, the contact angle of the substrate surface decreased to 60.4°, which is due to the large number of amino groups in ATMS, making the substrate surface hydrophilic. When spiropyran was grafted to the substrate surface through the reaction of the amino group and the carboxyl group, the contact angle reached 103.4°, because the closed-ring SP form of spiropyran is hydrophobic. Finally, under UV irradiation, the spiropyran grafted on the substrate isomerized to the zwitterionic MC form, showing a hydrophilic property, so the contact angle was only 37.0° [33]. The change in the contact angle was consistent with the functionalization of the substrate surface in each step, which fully proved the successful preparation of the composite substrate.

### 3.3. Optimization of the Preparation Process of PDMS/PMMA-Au/SP Composite Substrate

#### 3.3.1. Optimization of Au Nanoparticle Concentration

**Figure 4:**
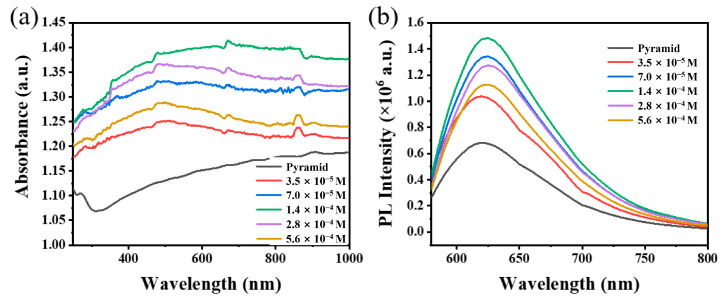
(**a**) Absorption spectra and (**b**) fluorescence spectra of PDMS/PMMA-Au/SP with different Au nanoparticle concentrations in PMMA solution at spin coating process.

Firstly, the effect of Au nanoparticles’ concentration on the fluorescence enhancement performance of the PDMS/PMMA-Au/SP composite substrate was studied. The composite substrates discussed below were all treated with UV light for 90 s. As shown in Figure 4b, when there were no Au nanoparticles, the fluorescence intensity of the composite substrate was very weak under incident light excitation. The fluorescence intensity of the composite substrate increased with an increase in the concentration of Au nanoparticles, which is caused by the plasmon effect of Au nanoparticles [19]. When the concentration of Au nanoparticles was 1.4 × 10^−4^ M, the fluorescence intensity of the composite substrate reached a larger value, and the corresponding plasmon resonance absorption peak was stronger (Figure 4a). However, as the concentration of Au nanoparticles continued to increase, the fluorescence intensity decreased, because accumulation of the Au nanoparticles will occur when the concentration of Au nanoparticles is too high, which weakens the electromagnetic field effect.

#### 3.3.2. Optimization of TBDS Addition

**Figure 5:**
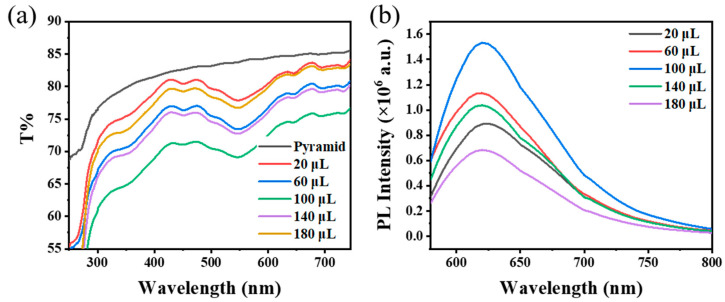
(**a**) Transmission spectra and (**b**) fluorescence spectra of PDMS/PMMA-Au/SP composite substrates with different levels of TBDS addition in the preparation process.

Secondly, the effect of TBDS addition on the fluorescence enhancement performance of the PDMS/PMMA-Au/SP composite substrate was studied. The composite substrates discussed below were all treated with UV light for 90 s. As shown in Figure 5, when the additive amount of TBDS was 100 μL, the transmittance of the composite substrate was lower; that is, the discoloration of spiropyran on the surface of the composite substrate was more obvious, and the fluorescence intensity reached a larger value. This is because the density of TBDS on the surface of the substrate will affect the spacing between spiropyrans and further affect the steric hindrance in the ring-opening isomerization process of spiropyran. When the density of TBDS was too low, the spacing between the spiropyran molecules was too small for their isomerization, resulting in the slow or even impossible ring opening of spiropyran, which led to small changes in the composite substrate’s transmittance and weak fluorescence intensity. However, if the density of TBDS was too high, the amount of spiropyran on the surface of the composite substrate would be reduced, and the change in the transmittance and fluorescence intensity of the composite substrate would be small.

#### 3.3.3. Optimization of ATMS Addition

**Figure 6:**
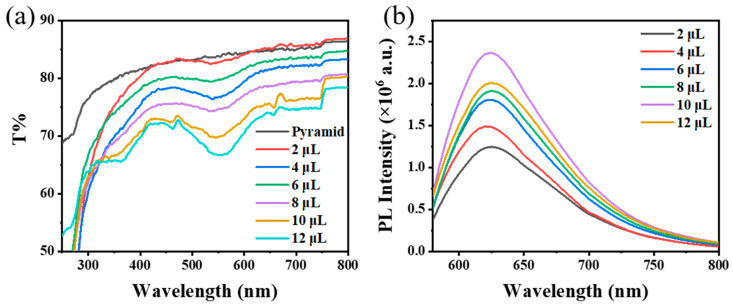
(**a**) Transmission spectra and (**b**) fluorescence spectra of PDMS/PMMA-Au/SP composite substrates with different levels of ATMS addition in the preparation process.

Then, the effect of ATMS addition on the fluorescence enhancement performance of the PDMS/PMMA-Au/SP composite substrate was studied. The composite substrates discussed below were all treated with UV light for 90 s. The additive amount of ATMS will affect the number of graft sites for spiropyran on the substrate surface. As shown in Figure 6, when the additive amount of ATMS was too small, graft sites on substrate surface were not enough, resulting in low spiropyran grafting amounts, and the characteristic peak of spiropyran in the transmittance spectrum became small and the fluorescence intensity of the substrate was weak. With an increase in ATMS addition, the transmittance of the composite substrate continued to decrease, and the fluorescence intensity gradually increased. The fluorescence intensity of the composite substrate was higher when the additive amount of ATMS was 10 μL. However, the fluorescence intensity of the composite substrate decreased when the addition of ATMS exceeded 10 μL, which was because the density of spiropyran was too high, resulting in fluorescence quenching due to aggregation [34].

#### 3.3.4. Optimization of Spiropyran Grafting Time

**Figure 7:**
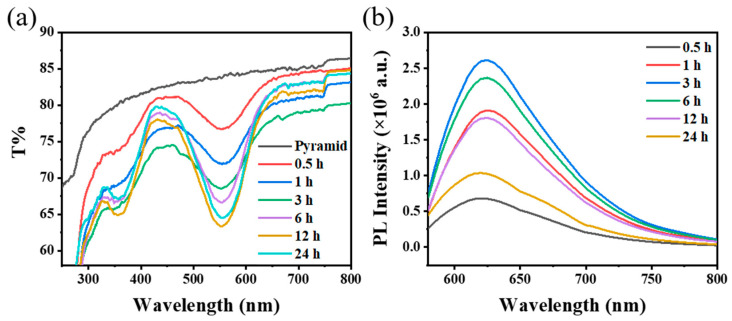
(**a**) Transmission spectra and (**b**) fluorescence spectra of PDMS/PMMA-Au/SP composite substrates with different spiropyran grafting times in the preparation process.

Finally, the effect of the spiropyran grafting time on the fluorescence enhancement performance of the PDMS/PMMA-Au/SP composite substrate was studied, as shown in Figure 7. The composite substrates discussed below were all treated with UV light for 90 s. The grafting time of spiropyran was closely related to the grafting amount of spiropyran on the composite substrate surface. When the grafting time was too short, the number of spiropyran molecules grafted on the substrate was less, so the transmission spectrum of the composite substrate changed less. With an increase in grafting time, the transmittance of the composite substrate decreased, and the fluorescence intensity increased gradually. When the grafting time was 12 h, the transmittance of the composite substrate changed most obviously. However, it should be noted that the fluorescence intensity of the composite substrate reached a large value when grafting time was 3 h and then showed a decreasing trend with a further increase in grafting time. This may be due to the aggregation quenching of spiropyran causing by the increase in the density of spiropyran on the substrate when the grafting time was longer than 3 h. Here, we chose a spiropyran grafting time of 3 h as the optimal condition for the follow-up study because although the transmittance peak of the composite substrate was 3% lower when the grafting time increased from 3 h to 12 h, the fluorescence intensity was reduced by about 30%. Here, the composite substrate is intended to be used for the subsequent trace detection of metal ions, so excellent fluorescence performance is very important for the practical application of this substrate.

### 3.4. Fluorescence Enhancement Property of PDMS/PMMA-Au/SP Substrate

**Figure 8:**
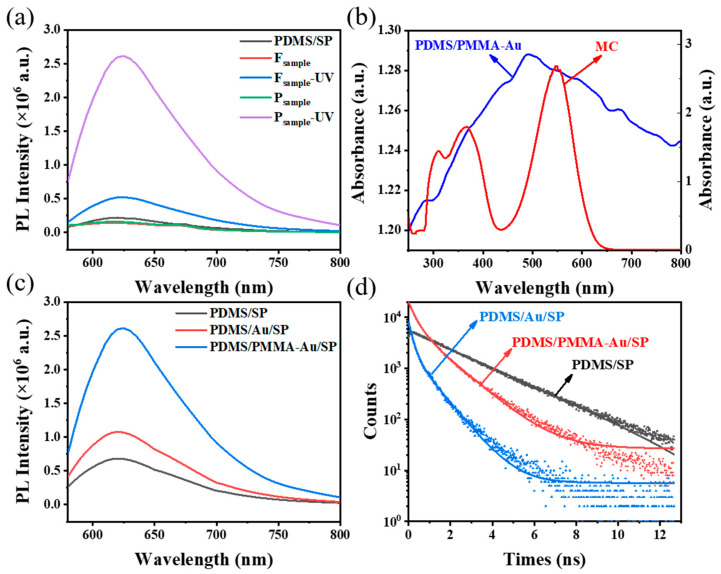
(**a**) Fluorescence spectra of substrate without inverted pyramid structure and substrate with inverted pyramid structure before and after UV illumination. (**b**) Absorption spectra of PDMS/PMMA-Au (black) and MC ethanol solution (red). (**c**) Fluorescence spectra of PDMS/SP (black), PDMS/Au/SP (red) and PDMS/PMMA-Au/SP (blue). (**d**) Time-resolved emission spectra of PDMS/SP (black), PDMS/Au/SP (blue) and PDMS/PMMA-Au/SP (red).

In the process of single-factor optimization, we have demonstrated that the PDMS/PMMA-Au/SP substrate exhibited an excellent fluorescence property when the grafting time of spiropyran was 3 h. The fluorescence enhancement mainly came from three aspects: good anti-reflection ability of the inverted pyramid structure, plasmon resonance effect of Au nanoparticles and low probability of non-radiative energy transfer realized by the PMMA isolation layer. (1) The inverted pyramid structure had excellent anti-reflection ability, which could improve the excitation efficiency of spiropyran on the substrate surface, and thus improve its fluorescence intensity. To prove this, the fluorescence intensity of the P_sample_ (PDMS/PMMA-Au/SP) with an inverted pyramid structure and the F_sample_ without an inverted pyramid structure was compared, as shown in Figure 8a. The fluorescence intensity of the former was 19 times that of the latter, and the enhancement factor was as high as 5.06. (2) Au nanoparticles could generate an enhanced local electromagnetic field under incident light, so as to provide more rapid attenuation channels for spiropyran on the substrate surface, reduce its fluorescence lifetime [35], increase its spontaneous radiation rate and enhance its fluorescence intensity. To prove this, the fluorescence intensity and fluorescence lifetime of PDMS/PMMA-Au/SP with Au nanoparticles and PDMS/SP without Au nanoparticles were compared. The fluorescence intensity of PDMS/PMMA-Au/SP was significantly higher than that of PDMS/SP, and the fluorescence lifetime of PDMS/PMMA-Au/SP was shorter than that of PDMS/SP, as shown in Figure 8c,d. In addition, the resonance coupling between Au nanoparticles and fluorescent molecules could significantly enhance the fluorescence intensity of the latter, and this enhancement effect reached its maximum when the plasmon resonance peak of the metal nanoparticles overlapped with the absorption or excitation peak of the fluorescent molecules [36]. As shown in Figure 8b, the plasmon resonance absorption peak of the Au nanoparticles overlapped with the absorption peak of spiropyran to a high degree, which would significantly improve the excitation efficiency and radiation rate of spiropyran on the substrate surface, and thus improve its fluorescence intensity. (3) The design of the PMMA isolation layer reduced the probability of non-radiative energy transfer caused by direct contact between Au and MC, thereby increasing the fluorescence intensity of the substrate. To prove this, the fluorescence intensity and fluorescence lifetime of the substrate containing PMMA (PDMS/PMMA-Au/SP) and the substrate without PMMA (PDMS/Au/SP) were compared, as shown in Figure 8c,d. The fluorescence intensity of PDMS/PMMA-Au/SP was higher than that of PDMS/Au/SP, and the fluorescence lifetime of PDMS/PMMA-Au/SP was longer than that of PDMS/Au/SP, indicating that there was more electron transfer between the metal nanoparticles and the spiropyrans on PDMS/Au/SP, which led to the fluorescence quenching of MC.

### 3.5. Metal Ion Detection

**Figure 9:**
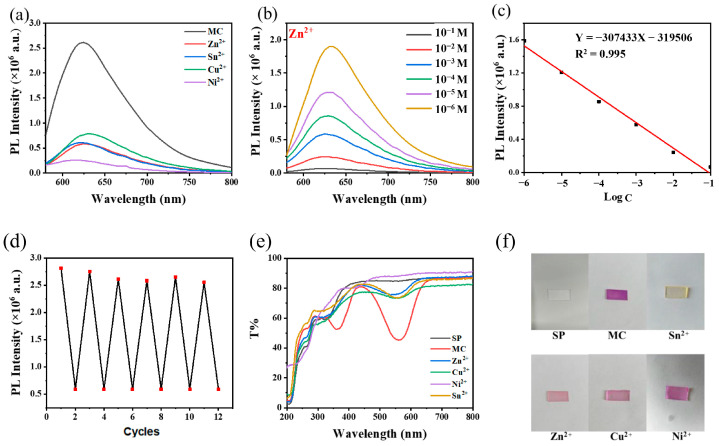
(**a**) Fluorescence spectra of PDMS/PMMA-Au/SP substrate when it comes into contact with solutions of different metal ions (10^−3^ M in ethanol) after UV irradiation. (**b**) Fluorescence spectra of PDMS/PMMA-Au/SP substrate when it comes into contact with Zn^2+^ ethanol solution of different concentrations after UV irradiation. (**c**) Linear plots of fluorescence intensity versus the logarithm of Zn^2+^ concentration. (**d**) Cycle test for PDMS/PMMA-Au/SP substrate in Zn^2+^ detection. (**e**) Transmission spectra of PDMS/PMMA-Au/SP substrate when it comes into contact with solutions of different metal ions (10^−3^ M in ethanol) after UV irradiation. (**f**) The real picture of PDMS/PMMA-Au/SP substrate when it comes into contact with solutions of different metal ions (10^−3^ M in ethanol) after UV irradiation.

When spiropyran isomerizes from the SP form to the MC form under UV irradiation, the fluorescence and colorimetric sensing of metal ions can be realized through the complexation of MC’s phenoxy group with metal ions. As shown in Figure 9a, when a 10^−3^ M ethanol solution of Zn^2+^, Ni^2+^, Sn^2+^ and Cu^2+^ was added to the surface of the PDMS/PMMA-Au/SP substrate, the fluorescence peak of MC shifted from 620 nm to 615 nm, 610 nm, 625 nm and 630 nm respectively, and the fluorescence of MC was obviously quenched. The fluorescence intensity of the PDMS/PMMA-Au/SP substrate was linear with the concentration of metal ions. Taking Zn^2+^ as an example to show the ability of PDMS/PMMA-Au/SP in metal ion sensing, the reason for choosing Zn^2+^ is that it plays an important role in many physiological and pathological processes, and identifying Zn^2+^ is of great theoretical and practical significance. As shown in Figure 9c, the linear analysis equation of substrate fluorescence intensity and Zn^2+^ concentration was as follows: Y = −307433X − 319506 (10^−1^ M~10^−6^ M), and the detection limit (LOD) was 0.281 μM (LOD = 3S_b_/S, S_b_ = 0.08613), which indicated that the substrate could be used for the trace detection of metal ions. In addition, since the complex bond formed between MC and metal ions was unstable, when the substrate was exposed to visible light, spiropyran would isomerize back to the close-ring state [37], which made the substrate recyclable. The PDMS/PMMA-Au/SP substrate did not show obvious attenuation of sensitivity within six cycles (Figure 9d). Si_ph_/TiO_2_/PMMA-SP/Au@SiO_2_, reported in our previous work, showed lower LOD (0.054 μM) to Zn^2+^ due to Au/Ag-coupled plasmonic resonance, but it is a hard substrate and cannot realize the visual identification of metal ions. Here, PDMS/PMMA-Au/SP is a soft substrate and can also detect different metal ions by colorimetry. Figure 9e,f show the real picture and transmission spectra of the PDMS/PMMA-Au/SP substrate in response to different metal ions. After UV irradiation for 150 s, spiropyran on the substrate surface isomerized from the colorless SP form to the deep-purple MC form, resulting in an obvious color change of the substrate, which coincided with the transmission spectrum change of the substrate. When MC was complexed with different metal ions, its transmission peak showed an obvious blue shift, resulting in the color change of the substrate. In this way, different metal ions could be distinguished by the naked eye; this visual identification method greatly reduces the cost of ion detection. However, in the mixed solution of several metal ions, this substrate cannot be used for the specific recognition of each ion, which is expected to be further solved by the modification of the spiropyran.

## 4. Conclusions

PDMS soft substrates containing inverted pyramid microstructures were prepared by template-impressing techniques, then PMMA-Au thin films were assembled on the surface of the soft substrates. After that, a composite substrate of PDMS/PMMA-Au/SP was obtained by modifying tert-butyldiphenylchlorosilane (TBDS) and spiropyran on the surface of the PMMA-Au thin films by covalent grafting. The composite substrate was used for colorimetric and fluorescence sensing of metal ions. The fluorescence enhancement factor of the substrate compared to the open-ring MC form of spiropyran was as high as 5.06, and its fluorescence detection limit for Zn^2+^ was 0.281 μM. Moreover, the color transformation of the composite substrate from colorless to deep purple can be completed within 150 s, and the visual identification of metal ions such as Zn^2+^, Ni^2+^, Sn^2+^ and Cu^2+^ can be realized.

## Data Availability

Not applicable.
